# Erlotinib Preserves Renal Function and Prevents Salt Retention in Doxorubicin Treated Nephrotic Rats

**DOI:** 10.1371/journal.pone.0054738

**Published:** 2013-01-18

**Authors:** Raed N. Bou Matar, Janet D. Klein, Jeff M. Sands

**Affiliations:** 1 Department of Pediatric Medicine, Emory University, Atlanta, Georgia, United States of America; 2 Children's Healthcare of Atlanta, Emory University, Atlanta, Georgia, United States of America; 3 Renal Division, Department of Medicine, Emory University, Atlanta, Georgia, United States of America; 4 Department of Physiology, Emory University, Atlanta, Georgia, United States of America; Aarhus University, Denmark

## Abstract

Nephrotic syndrome is associated with up-regulation of the heparin-binding epidermal growth factor (HB-EGF). Erlotinib blocks the activation of the epidermal growth factor receptor (EGFR) in response to HB-EGF. This study investigates the effect of Erlotinib on the progression of proteinuria, renal dysfunction, and salt retention in doxorubicin treated nephrotic rats. Male rats were divided into 3 pair-fed groups (n = 13/group) as follows: Control rats (*Ctrl*); rats receiving intravenous doxorubicin (*Dox*); and rats receiving intravenous doxorubicin followed by daily oral Erlotinib (*Dox + Erl*). Upon establishment of high grade proteinuria, urine sodium and creatinine clearance were measured. Kidney tissue was dissected and analyzed for γ-epithelial sodium channel (γENaC), sodium-potassium -chloride co-transporter 2 (NKCC2), sodium chloride co-transporter (NCC), aquaporin 2 (AQP2), and EGFR abundances using western blot. Creatinine clearance was preserved in the *Dox + Erl* rats as compared to the *Dox* group (in ml/min: *Ctrl*: 5.2±.5, *Dox*: 1.9±0.3, *Dox + Erl*: 3.6±0.5). Despite a minimal effect on the degree of proteinuria, Erlotinib prevented salt retention (Urinary Na in mEq/d: *Ctrl*: 2.2±0.2, *Dox*: 1.8±0.3, *Dox + Erl*: 2.2±0.2). The cleaved/uncleaved γENaC ratio was increased by 41±16% in the *Dox* group but unchanged in the *Dox + Erl* group when compared to *Ctrl*. The phosphorylated EGFR/total EGFR ratio was reduced by 74±7% in the *Dox* group and by 77±4% in the *Dox + Erl* group. In conclusion, Erlotinib preserved renal function and prevented salt retention in nephrotic rats. The observed effects do not appear to be mediated by direct blockade of EGFR.

## Introduction

Focal segmental glomerulosclerosis (FSGS) accounts for 3.3% of all incident end-stage renal disease (ESRD) cases in the United States (reviewed in [1]). Currently available treatment options are scarce and generally disappointing as only 20% of the treated patients achieve a satisfactory remission [2].

Up-regulation of the heparin-binding epidermal growth factor-like growth factor (HB-EGF), a member of the EGF family, in glomerular epithelial cells has been documented in animal models of nephrotic syndrome [3], [4], streptozotocin-induced diabetes [5], and rapidly progressive glomerulonephritis (RPGN) [6], [7]. The role HB-EGF plays in the development of glomerulosclerosis and renal failure in those diseases remains unclear. However, HB-EGF appears to promote renal epithelial cell repair, proliferation, and regeneration in the early stages of recovery after acute renal injury [8]. It also plays a role in the acute regulation of the glomerular filtration rate [7] and the immune-inflammatory reaction associated with RPGN [6]. Podocyte specific deletion of the epidermal growth factor receptor (EGFR) gene in mice prevents the development of crescentic glomerulonephritis and renal failure [6].

Erlotinib is a selective reversible inhibitor of the EGFR tyrosine kinase [9]. It inhibits autophosphorylation through binding to the adenosine triphosphate binding site of the receptor [9] and subsequently induces cytotoxicity in cancer cells through induction of reactive oxygen species [10]. It is FDA approved in the United States for the treatment of advanced non-small cell lung cancer and pancreatic cancer [11], [12]. Adverse effects are usually mild and include skin rash, diarrhea, fatigue, and rarely, interstitial pneumonitis [13], [14]. Erlotinib showed promise in the treatment of RPGN in early animal studies. It improves the course of RPGN, even when started 4 days after the induction of a mouse model of the disease [6]. In addition, other EGFR inhibitors were shown to reduce kidney enlargement [15], attenuate albuminuria and preserve podocyte structure in diabetic rats [16]. Interestingly, a monoclonal antibody against HB-EGF increases early albuminuria in the puromycin rat model of nephrotic syndrome [4].

Doxorubicin nephropathy in rats is an established model utilized to study the underlying pathophysiology of progressive FSGS [17]. As opposed to the reversible podocyte injury inflicted by puromycin, doxorubicin exerts a more severe initial cytotoxic podocyte injury followed by a delayed self-perpetuating progressive phase [18]. One advantage of this model is the ability to precisely control the timing of the initial glomerular injury, allowing the introduction of targeted therapy at a previously specified time period following the initial injury [18]. This also allows monitoring the progression of renal disease over a relatively longer duration after the initial injury (several weeks).

Experimental evidence suggests a major role of the epithelial sodium channel (ENaC) in the development of nephrotic syndrome associated salt and water retention [19]–[21]. In response to the activation of ENaC, we and others have observed a compensatory decrease in the abundance of the ascending limb and distal convoluted tubule sodium transporters, possibly mediated by reduced glomerular filtration rate [22]. As a result, conventional diuretics, which act primarily by inhibiting sodium transporters in the thick ascending limb of loop of Henle and distal convoluted tubules, are generally ineffective in nephrotic syndrome associated salt retention [23].

ENaC is regulated by various epidermal growth factors. Chronic treatment with TGF-α or EGF inhibits ENaC by decreasing the number of channels in the membrane, while acute treatment has a stimulatory effect [24]. The role, if any, which HB-EGF plays in the regulation of ENaC is not known. It also remains to be determined whether blockade of HB-EGF would have a direct regulatory effect on the nephrotic syndrome driven hyperactivity of ENaC.

We postulate that blockade of HB-EGF, through administration of oral Erlotinib, attenuates glomerular injury, proteinuria, and preserves renal function in the doxorubicin-induced rat model of nephrotic syndrome. We also postulate that Erlotinib promotes salt excretion in nephrotic rats through reduced abundance of the cleaved (and therefore active) form of γ-ENaC. Since increased ENaC activity has been associated with a compensatory reduction in the abundance of other tubular sodium transporters and aquaporin 2 (AQP2) [22], we postulate that HB-EGF blockade also preserves the abundance of the sodium-potassium-chloride co-transporter (NKCC2), the sodium-chloride co-transporter (NCC), and AQP2. Accordingly, we tested the effect of oral Erlotinib on the progression of proteinuria, renal dysfunction, and salt retention in pair-fed rats with doxorubicin-induced nephrotic syndrome. Changes in the tissue abundance of total EGFR, phosphorylated EGFR, γENaC subunit, AQP2, NKCC2, and NCC were also assessed.

## Methods

### Animals

The Emory University Institutional Animal Care and Use Committee approved all the protocols used in this study (IACUC Protocol 2001284) in strict accordance with the recommendations in the Guide for the Care and Use of Laboratory Animals of the National Institutes of Health. Three cohorts of male Sprague-Dawley rats were divided each into 3 weight matched groups (n = 13 total/group; Charles River Laboratories, Wilmington, MA) as follows: the first group was untreated and used as controls (*Ctrl*); the second group was anesthetized using isoflurane and received a sterile solution of intravenous (IV) doxorubicin (7.5 mg/kg body weight) once via the femoral vein (*Dox*); the third group received the same dose of IV doxorubicin in addition to oral Erlotinib 10 mg/kg daily mixed with powdered rat diet given starting at day 6 following the doxorubicin injection (*Dox + Erl*). The rats weighed 324±36 g (*Ctrl*), 321±34 g (*Dox*), and 320±40 g (*Dox + Erl*). Rats were housed singly and pair-fed. For pair-feeding, each *Ctrl* rat was paired with a weight-matched *Dox* rat and a *Dox + Erl* rat. Accurate pair-feeding was achieved by daily measurement of food intake of each of the matched triplets. The lowest measured 24-hour food intake in each triplet is then offered to all three matched animals over the next 24 hours. Pair-feeding was started 5 days following doxorubicin injection and maintained throughout the three week observation period. Regular rodent diet (LabDiet® 5001, PMI nutrition) containing 23.4 g % protein and 0.4 g % sodium was used for all groups. Rats were placed in metabolic cages throughout the observation period to allow for daily monitoring of food intake, water intake, and urine output. Animals were observed over a median period of 21 days (range 19–28 days) until reaching their primary endpoint. At that time, final urine samples were collected and the animals were killed by decapitation. Trunk blood was collected in red-top tubes (no additives), clotted at 4°C and centrifuged at 4000×g for 15 min. to pellet the red cells. Serum was collected and either analyzed immediately or stored frozen at −80°C. Kidneys were removed and dissected into inner medullary tip, inner medullary base, outer medulla and cortex tissue.

### Western Blot

Tissue samples of equal total protein concentration were prepared and size separated using SDS-PAGE on 7.5%, 10%, or 12.5% polyacrylamide gels. Proteins were then electroblotted to polyvinylidene difluoride membranes (Immobilon, Millipore, Bedford, MA), and analyzed as previously described [25]. Primary antibodies included antibodies to EGFR and phosphorylated EGFR (Cell Signaling, Danvers, MA), γENaC subunit [22], [26], AQP2 [25], [27], and NKCC2 [25], [27], [28]. Bands were visualized using infrared detection with the LICOR Odyssey protein analysis system and densitometry was performed with the same instrument (LICOR, Lincoln, NE). Data were normalized to the average densitometry of untreated animals in each group. Western blots were stained by Ponceau S to confirm equal loading of the gel.

### Laboratory analysis

Urine and serum creatinine were analyzed utilizing a Nova Biomedical blood gas analyzer. Urine sodium measurements were obtained using a sodium selective electrode (Cole-Parmer, Illinois). Total daily urine protein excretion was determined by Bio-Rad DC protein assay (Hercules, CA). Protein excretion was monitored in 24-hour urine samples every three to four days. Similarly, 24-hour urinary excretion of sodium was calculated by multiplying the urine sodium by urine flow rate. Pair-feeding ensured equal dietary sodium intake for the three groups of animals throughout the three-week observation period. Stool and sweat related sodium losses were considered negligible and therefore excluded from the estimation of daily sodium excretion. Glomerular filtration rate was estimated using creatinine clearance, calculated from the urine creatinine concentration, serum creatinine, and urine flow rate. Fractional excretion of water was calculated as the ratio of serum creatinine to urine creatinine.

### Statistical Analysis

A one-way ANOVA was used to analyze the differences between the three pair-fed groups of rats when a single category divided the observations, followed by Fisher's least significant difference (protected t-test), using GB Stat software (Dynamic Microsystems, Inc). When more than one category divided the observations, a two-way ANOVA was used. A value of p<0.05 was considered statistically significant. Densitometry data are presented as percentage change ± standard error [22].

## Results

**Figure 1:**
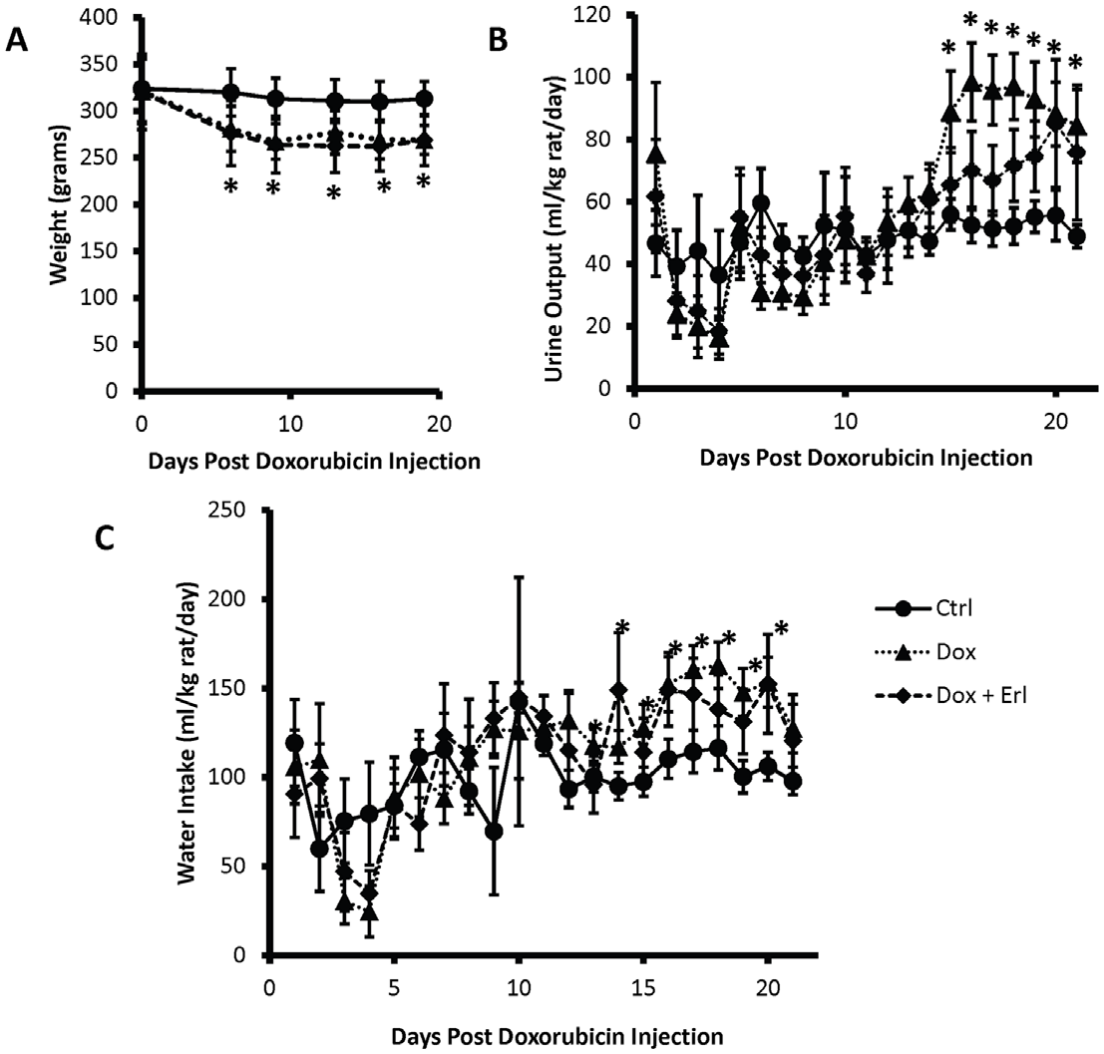
Doxorubicin-induced increases in water intake and urine output are reversed by Erlotinib treatment. Shown are the changes in weight (**A**), urine output (**B**), and water intake (**C**) plotted over time in control rats (solid line), doxorubicin (Dox) treated rats (dotted line), and doxorubicin + Erlotinib (Dox + Erl) treated rats (dashed line). Data are expressed as means ± SE (n = 13, * p<0.05 for Dox or Dox+Erl vs. control at same time point, and for Dox or Dox+Erl vs. Dox or Dox+Erl at time 0).

Baseline weight, age, and protein excretion were similar between the 3 groups analyzed. *Dox* rats and *Dox + Erl* rats developed a similar degree of weight loss during the first week of the experiment (*Dox* 12±8%, *Dox* + Erl 14±11%). Following this initial weight loss, all 3 groups of rats maintained a relatively steady weight over the remainder of the observation period (Figure 1A). Starting at day 15 following doxorubicin injection, rats in the *Dox* group developed an increase in water intake and urine output when compared to the *Controls* and *Dox* + Erl goups (Figures 1B and 1C). We observed upon sacrifice that there was a visible tissue swelling and ascites in the majority of nephrotic animals, closely resembling the generalized edema that occurs in human nephrotic syndrome.

### Urine protein, sodium and creatinine clearance

**Figure 2:**
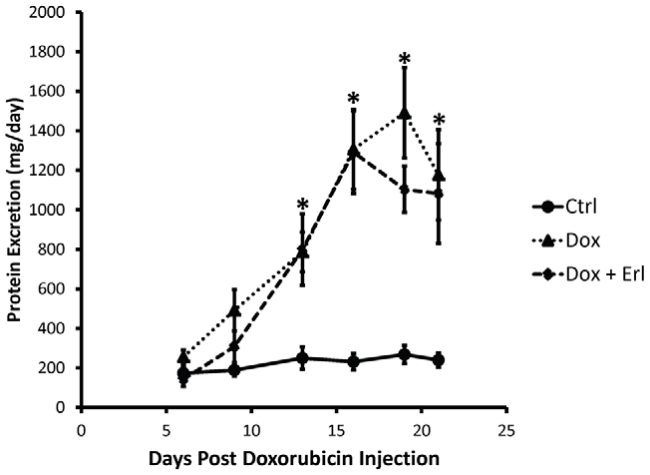
Proteinuria is apparent by 10 days after doxorubicin. The lines show the progression of the proteinuria plotted over time in control rats (solid line), doxorubicin treated rats (dotted line), and doxorubicin + Erlotinib treated rats (dashed line). Data are expressed as means ± SE (n = 13, * p<0.05 for Dox or Dox+Erl vs. control at same time point).

**Figure 3:**
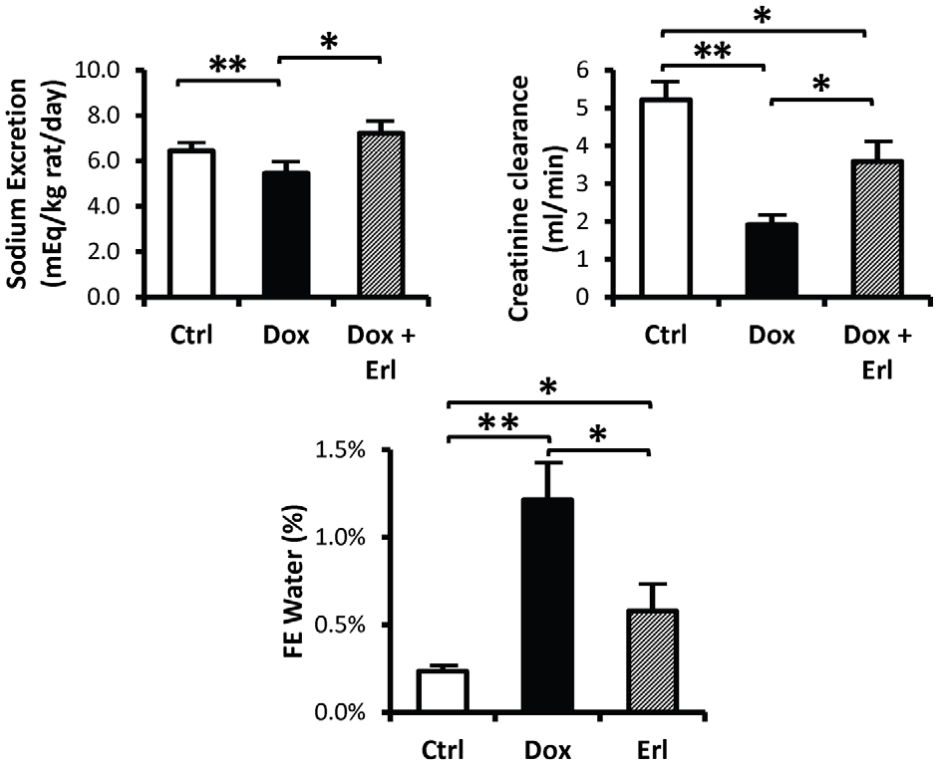
Erlotinib restores urine sodium excretion to control levels but only partially corrects creatinine clearance. **Left:** Bar graph showing the average urine sodium excretion evaluated at the end of the observation period in control rats (*Ctrl*; white bar), doxorubicin treated rats (*Dox*; solid black bar), and doxorubicin + Erlotinib treated rats (*Dox + Erl*; patterned bar). **Right:** Bar graph showing the average creatinine clearance evaluated at the end of the observation period in control rats (*Ctrl*; white bar), doxorubicin treated rats (*Dox*; solid black bar), and doxorubicin + Erlotinib treated rats (*Dox + Erl*; patterned bar). **Bottom**: Bar graph showing the average fractional excretion of water (FE Water) evaluated at the end of the observation period in control rats (*Ctrl*; white bar), doxorubicin treated rats (*Dox*; solid black bar), and doxorubicin + Erlotinib treated rats (*Dox + Erl*; patterned bar). Data are expressed as means ± SE (n = 13, * p<0.05, ** p<0.001).

All rats treated with doxorubicin developed a progressive increase in urinary protein excretion starting at day 9 following injection of doxorubicin. Urinary protein excretion was similar between the *Dox* and the *Dox* + Erl groups of animals, with a peak average protein excretion of 5.6 fold increase at day 16 in the *Dox* + Erl group and a peak average of 5.6 fold increase at day 19 in the *Dox* group (Figure 2). Urinary sodium excretion was reduced by 15±8% in the *Dox* group of rats when compared to control animals, but unchanged in *Dox* + Erl group. Creatinine clearance was reduced by 63±6% in the *Dox* group, but by only 31±10% in the *Dox* + Erl group of rats. The fractional excretion of water was increased by 5.2 fold in the *Dox* group when compared to controls, but by only 2.5 fold in the *Dox + Erl* group (Figure 3).

### EGFR and Phosphorylated EGFR

**Figure 4:**
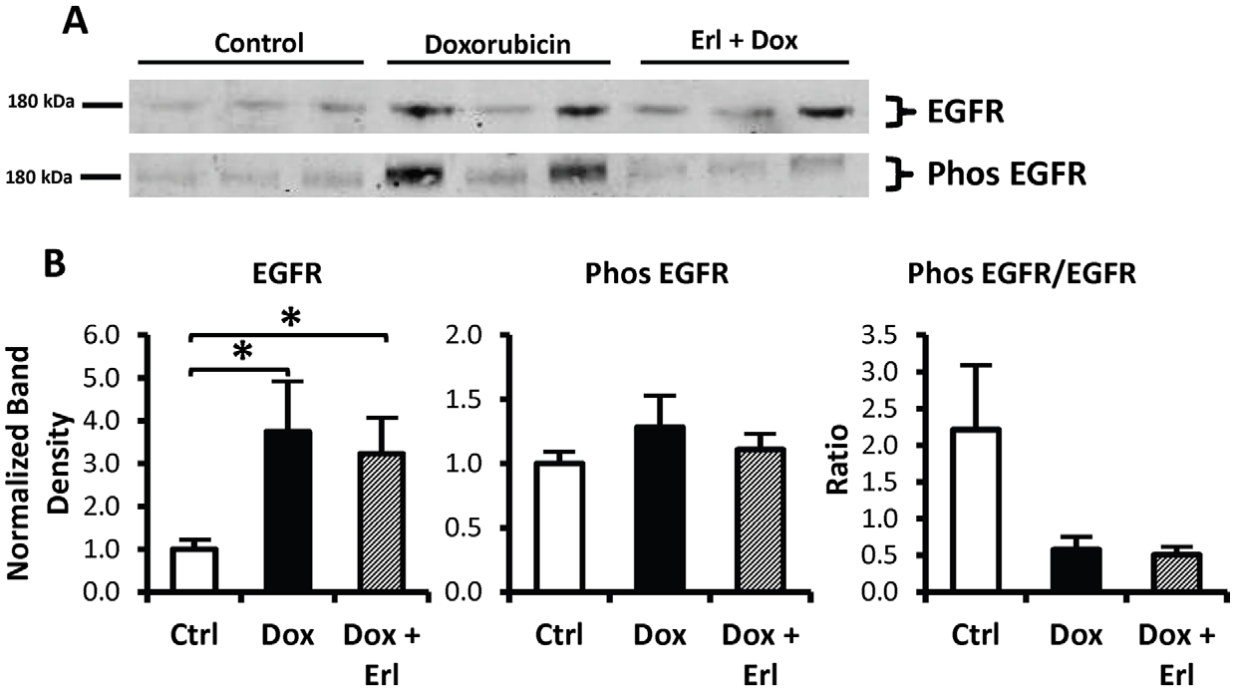
The doxorubicin-induced decrease in phosphorylated EGRF/EGRF ratio is not corrected by Erlotinib. EGFR and phosphorylated EGFR protein abundances in rat cortex tissue of control rats (*Ctrl*; white bars), doxorubicin treated rats (*Dox*; solid black bars), and doxorubicin + Erlotinib treated rats (*Dox + Erl*; patterned bars). **A**: Representative immunoblots showing abundances of EGFR and phosphorylated EGFR in rat cortex tissue lysates. An equal amount of total protein from a different rat tissue sample was loaded into each lane. **B**: Densitometric analysis of western blots from 3 cohorts of animals. Data were normalized for the average densitometry of untreated animals in each group. **Right:** Total EGFR densitometric analysis. **Middle:** Phosphorylated EGFR densitometric analysis. **Left:** The ratio of phosphorylated EGFR to total EGFR densitometries. Data are expressed as means ± SE (n = 11, * p<0.05).

Cortex tissue abundance of EGFR was increased by 2.7 fold and 2.2 fold in the *Dox* and *Dox + Erl* group of rats, respectively. Phosphorylated EGFR abundance in cortex tissue was not significantly different in the groups analyzed. The ratio of phosphorylated EGFR to total EGFR was reduced by 74±7% in the *Dox* group and by 77±4% in the *Dox + Erl* group (Figure 4).

### γENaC

**Figure 5:**
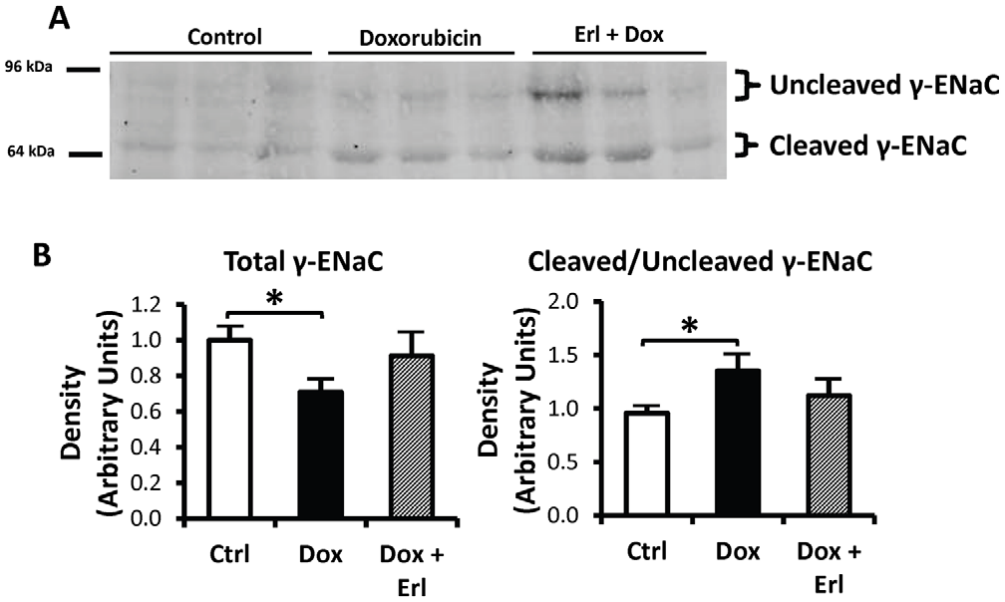
Cleaved γENaC subunit protein abundance is increased by doxorubincin relative to control or Erlotinib-treated animals. Shown is the γENaC subunit in rat cortex tissue of control rats (*Ctrl*; white bars), doxorubicin treated rats (*Dox*; solid black bars), and doxorubicin + Erlotinib treated rats (*Dox + Erl*; patterned bars). **A**: Representative immunoblots showing abundance of γENaC in rat cortex tissue lysates. Each lane was loaded with an equal amount of total protein from a different rat. **B**: Densitometric analysis of western blots from 3 cohorts of animals. Data were normalized for the average densitometry of untreated animals in each group. Separate densitometric analysis was performed for the total, cleaved, and uncleaved γENaC. **Left:** Total ENaC densitometric analysis. **Right:** The ratio of cleaved to uncleaved γENaC densitometries. Data are expressed as means ± SE (n = 11, * p<0.05).

The total cortex tissue abundance of γENaC was reduced by 45±9% in the *Dox* group of rats when compared with controls, whereas levels in the *Dox* + Erl group were not different from control levels. The difference in the cortex tissue abundance of γENaC between the *Dox* and the *Dox + Erl* groups of rats did not reach statistical significance. The ratio of cleaved to uncleaved cortex tissue abundance of γENaC was increased by 41±16% in the *Dox* group when compared to controls, but unchanged in the *Dox + Erl* group. The difference in cleaved to uncleaved γENaC ratio between the *Dox* and *Dox + Erl* groups did not reach statistical significance (Figure 5).

### NKCC2 and NCC

**Figure 6:**
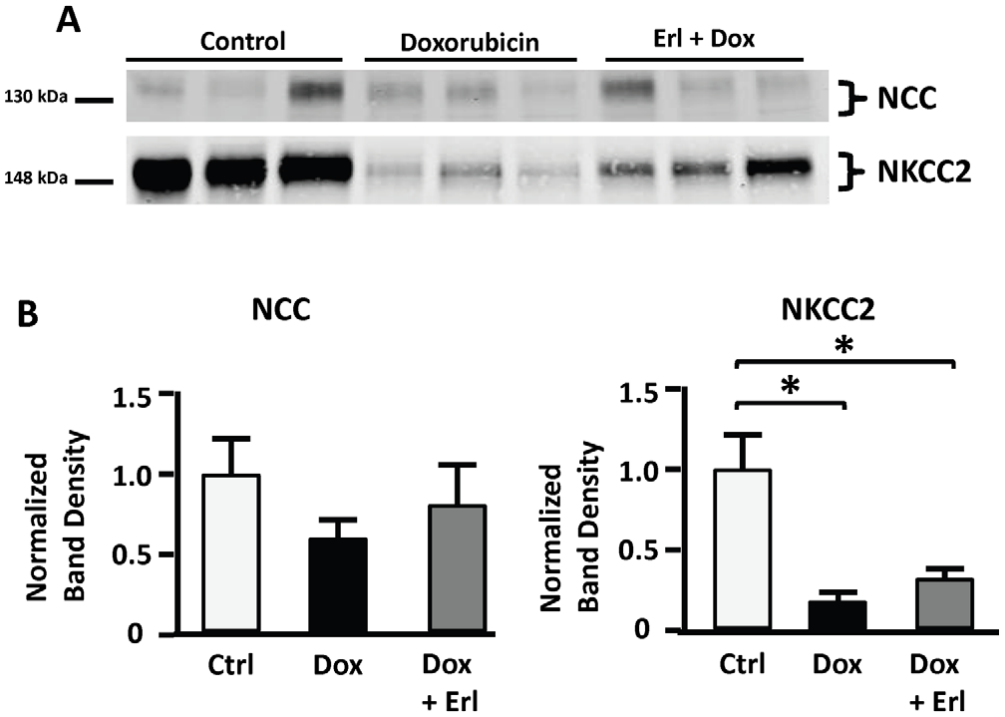
NCC and NKCC2 show different responses to doxorubicin and Erlotinib. Shown are NCC and NKCC2 protein abundances in cortex and outer medullary tissue of control rats (*Ctrl*; white bars), doxorubicin treated rats (*Dox*; solid black bars), and doxorubicin + Erlotinib treated rats (*Dox + Erl*; patterned bars). **A**: Representative immunoblot showing abundance of NCC in cortex tissue lysates and NKCC2 in outer medullary tissue lysates. An equal amount of total tissue lysate protein from a different rat was loaded into each lane. **B**: Densitometric analysis of western blots from 3 cohorts of animals. Data were normalized for the average densitometry of untreated animals in each group. Data are expressed as means ± SE (n = 11, * p<0.05).

NKCC2 abundance in the outer medulla was reduced by 78±8% in the *Dox* group and by 71±8% in the *Dox + Erl* group of animals. Cortex tissue abundance of NCC was not significantly different in any of the groups analyzed (Figure 6).

### AQP2

**Figure 7:**
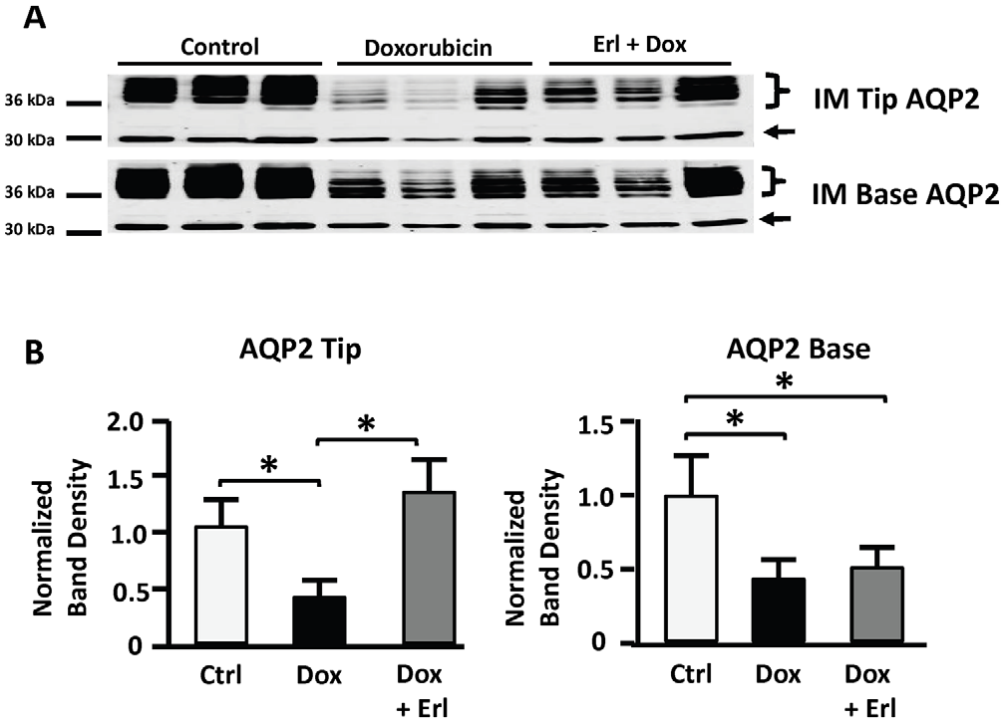
Doxorubicin decreases AQP2 in inner medulla (IM) and Erlotinib restores levels in IM tip. Shown: AQP2 protein abundance in IM tip and base tissue of control rats (*Ctrl*; white bars), doxorubicin treated rats (*Dox*; solid black bars), and doxorubicin + Erlotinib treated rats (*Dox + Erl*; patterned bars). **A**: Representative immunoblots showing abundance of AQP2 in IM tip and base tissue lysates. Each lane containes samples from a different rat. **B**: Densitometric analysis of western blots from 3 cohorts of animals. Data were normalized for the average densitometry of untreated animals in each group. Data are expressed as means ± SE (n = 11, * p<0.05).

AQP2 abundance was reduced by 60±12% in inner medullary tip tissue of the *Dox* group of rats when compared to control*s*, but remained unchanged in the *Dox + Erl* group. In inner medullary base tissue, AQP2 abundance was reduced by 70±6% and 61±7% in the *Dox* and *Dox + Erl* groups, respectively, when compared to controls (Figure 7).

## Discussion

In rats with doxorubicin induced nephrotic syndrome, oral Erlotinib, started at day 6 following the initial glomerular injury, had no effect on the course of proteinuria, but resulted in improved renal water handling, reversal of salt retention, and a partial preservation of renal function. This was mediated by conserved AQP2 abundance in the inner medullary tip of Erlotinib treated rats as compared to their untreated nephrotic counterparts. Erlotinib did not alter the decrease in AQP2 abundance in the inner medullary base tissue of nephrotic rats. NKCC2 protein abundance was similarly reduced in the outer medulla of both Erlotinib treated and untreated nephrotic rats. EGFR protein abundance was increased in both Erlotinib treated and untreated nephrotic rats. NKCC2, EGFR, and phosphorylated EGFR abundances were unaltered by Erlotinib treatment in nephrotic rats.

Reduced abundance of inner medullary AQP2 has been reported in various rodent models of nephrotic syndrome [22], [29]–[31]. In our study, AQP2 abundance appears to be preserved in Erlotinib treated nephrotic rats as compared to their untreated counterparts. This effect is consistent with improved water handling, as reflected by reduced urine output in Erlotinib treated animals. The effect is prominent in the inner medullary tip, but not in the inner medullary base tissue. The reasons behind this discrepancy are unclear. One possibility is that the decrease in AQP2 could be secondary to the disruption in the corticomedullary osmotic gradient that is expected in association with a profound reduction in the abundance of thick ascending limb NKCC2 in nephrotic rats [22], [27], [30], [32]. While a decrease in NKCC2 could lead to severe salt wasting as in Bartter's Syndrome, in nephrotic syndrome NKCC2 reduction is secondary to a state of salt retention. This helps explain why loop diuretics have a minimal effect in nephrotic salt retention (as the diuretic effect is mediated through blocking NKCC2).

The observed preservation of renal function in nephrotic rats is consistent with a recent report in a mouse model of RPGN [6], signifying that this benefit is not specific to RPGN or to mice, and may be generalized to any model of severe acute glomerular injury. Further studies are needed to evaluate the effect of Erlotinib on other models of acute glomerular injury, such as lupus nephritis or IgA nephropathy. In RPGN, the preservation of renal function was attributed to reduced phosphorylation of EGFR; however, we did not observe significant differences in the ratio of phosphorylated EGFR to total EGFR. Although our observations were made in whole cortical tissue lysate and thus cannot exclude possible localized effect of EGFR on the tubules, our results suggest that a detectable change in phosphorylated EGFR may not be necessary to achieve the beneficial effects or that the effects of Erlotinib are mediated through other, as yet undetermined, mechanistic pathways.

In an earlier study, a monoclonal antibody against HB-EGFR increased early albuminuria in the puromycin rat model of nephrotic syndrome [4]. The discrepancy between this result and our current study may be explained by differences in the underlying pathophysiology between the doxorubicin and puromycin models of nephrotic syndrome. Contrary to puromycin induced glomerular injury, the severity of the injury induced by doxorubicin generally exceeds the threshold of irreversible damage [18]. This leads to a steady progression to glomerulosclerosis and advanced renal insufficiency [18]. Hence, it is not surprising that HB-EGF blockade yields beneficial effects on the progression of renal insufficiency in the setting of doxorubicin (but not puromycin) induced nephrotic syndrome.

Our study is the first, to date, to evaluate the effect of Erlotinib on renal salt handling in nephrotic syndrome. The normalization of salt excretion in Erlotinib treated nephrotic rats cannot be simply explained by changes in the glomerular filtration rate. In our previous study, polyuria and polydipsia heralded salt retention and a concurrent defect in water handling in nephrotic rats [22]. Unlike their untreated counterparts, nephrotic rats treated with Erlotinib did not develop this phase of polyuria and polydipsia and had an attenuated increase in the fractional excretion of water. Direct activation of ENaC, through cleavage of the γ-subunit, has been suggested as a possible cause of nephrotic syndrome associated salt retention [19], [21], [22]. Therefore, our study tested the hypothesis that the Erlotinib effect on sodium excretion is mediated through reduced cleavage of γENaC. Despite a decrease in the abundance of total γENaC in *Dox* rats, we observed a significant increase in the cleaved to uncleaved proportion of γENaC. Based on previous in-vitro studies, cleavage of the γ-subunit is expected to “lock” ENaC in the open state, promoting avid reabsorption of sodium [19]–[21]. The ratio of cleaved to uncleaved γENaC in the Erlotinib treated nephrotic animals was not statistically decreased, although there was a tendency to normalization. The lack of statistical significance may be due to the animal to animal variability inherent in this type of study. Although our results did not reach statistical significance, the tendency towards a ratio comparable to that in control animals is consistent with the normalization of salt excretion observed in Erlotinib-treated nephrotic rats. As for other major transporters involved in renal salt handling, Erlotinib treatment did not alter the outer medullary tissue abundance of NKCC2 or the cortical abundance of NCC in nephrotic rats.

As is often the case with animal studies, our results are limited by the wide animal-to-animal variability, a common feature of many rodent models of nephrotic syndrome [18]. This variability was minimized by utilizing weight and age matched rats. To avoid any possible confounding effect of a nutritional bias, animals were pair fed throughout the 3-week observation period. Therefore, equal daily dietary sodium intake between the three groups was maintained throughout the study. We chose to use a dose of Erlotinib that was previously shown to be effective in ameliorating the progression of RPGN in a mouse model [6]. This dose was generally well tolerated in our study cohort. It is possible, however, that lower doses (1–2 mg/kg/day), similar to those used in treating lung cancer in humans, may provide a similar beneficial effect with a lower risk for toxicity. Further study is required to determine the optimal effective Erlotinib dose. Considering the relatively short half-life of doxorubicin (12–18.5 hours in humans), it appears unlikely that doxorubicin would continue to exert a direct effect on the tubules at the time of our analysis, 3 weeks following the initial drug infusion. Furthermore, multiple previous studies observed similar changes in tubular protein abundance in other rodent models of nephrotic syndrome [30], [31]. This suggests that the detected changes in tubular transporters are likely to be disease specific rather than model specific.

In summary, despite a minimal effect on the progression of proteinuria, Erlotinib reversed the reduction in AQP2 abundance in inner medullary base tissue, improved water handling as reflected by reduced urine output, prevented salt retention, and partially preserved renal function in doxorubicin-treated nephrotic rats. Erlotinib effects do not appear to be mediated by blockade of EGFR phosphorylation. Future studies are needed to test a potentially generalizable beneficial role for Erlotinib treatment in other forms of acute glomerular injury and to delineate the underlying mechanism(s) of action.
